# Development of an orthotopic syngeneic murine model of colorectal cancer for use in translational research

**DOI:** 10.1177/0023677219826165

**Published:** 2019-02-13

**Authors:** Jonathan P Evans, Boleslaw K Winiarski, Paul A Sutton, Lorenzo Ressel, Carrie A Duckworth, D Mark Pritchard, Daniel H Palmer, Christopher E Goldring, Neil R Kitteringham

**Affiliations:** 1 Department of Molecular and Clinical Cancer Medicine, University of Liverpool, UK; 2 Department of Molecular and Clinical Pharmacology, University of Liverpool, UK; 3 Department of Veterinary Pathology, University of Liverpool, UK; 4 Department of Cellular and Molecular Physiology, University of Liverpool, UK; 5 Clatterbridge Cancer Centre, Liverpool, UK

**Keywords:** colorectal cancer, orthotopic syngeneic model, bioluminescent imaging

## Abstract

Improving outcomes in colorectal cancer requires more accurate *in vivo* modelling of the disease in humans, allowing more reliable pre-clinical assessment of potential therapies. Novel imaging techniques are necessary to improve the longitudinal assessment of disease burden in these models, reducing the number of animals required for translational studies. This report describes the development of an immune-competent syngeneic orthotopic murine model of colorectal cancer, utilising caecal implantation of CT26 cells stably transfected with the luciferase gene into immune-competent BALB/c mice, allowing serial bioluminescent imaging of cancer progression. Luminescence in the stably transfected CT26 cell line, after pre-conditioning in the flank of a BALB/c mouse, accurately reflected cell viability and resulted in primary caecal tumours in five of eight (63%) mice in the initial pilot study following caecal injection. Luminescent signal continued to increase throughout the study period with one mouse (20%) developing a liver metastasis. Histopathological assessment confirmed tumours to be consistent with a poorly differentiated adenocarcinoma. We have now performed this technique in 68 immune-competent BALB/c mice. There have been no complications from the procedure or peri-operative deaths, with primary tumours developing in 44 (65%) mice and liver metastases in nine (20%) of these. This technique provides an accurate model of colorectal cancer with tumours developing in the correct microenvironment and metastasising to the liver with a similar frequency to that seen in patients presenting with colorectal cancer, with serial bioluminescent reducing the murine numbers required in studies by removing the need for cull for assessment of disease burden.

## Résumé

L'amélioration des résultats concernant le cancer colorectal (CCR) nécessite une modélisation in vivo plus précise de la maladie chez l'homme, ce qui permet une plus grande fiabilité d'évaluation pré-clinique des thérapies potentielles. De nouvelles techniques d'imagerie sont nécessaires pour améliorer l'évaluation longitudinale du fardeau de la maladie dans ces modèles, afin de réduire le nombre d'animaux requis pour les études translationnelles. Ce rapport décrit le développement d'un modèle murin orthotopique syngénique immuno-compétent de CCR, utilisant l'implantation cæcale de cellules CT26 transfectées de façon stable avec le gène de la luciférase dans les souris BALB/c immuno-compétentes, ce qui permet d'utiliser l'imagerie bioluminescente sérielle pour voir l'évolution du cancer. La luminescence des cellules CT26 transfectées de façon stable, après, pré-conditionnement, dans le flanc d'une souris BALB/c, a clairement reflété la viabilité cellulaire et a donné lieu à des tumeurs cæcales primaires chez 8 des 5 (63%) souris dans l'étude pilote initiale après injection cæcale. Le signal luminescent a continué d'augmenter tout au long de la période d'étude avec une souris (20%) développant des métastases hépatiques. L'évaluation histopathologique a confirmé que les tumeurs étaient compatibles avec un adénocarcinome peu différencié. Nous avons utilisé cette technique chez 68 souris BALB/c immuno-compétentes. Il n'y a pas eu de complications dues à la procédure ni de décès péri-opératoires, avec des tumeurs primaires chez 44 (65%) des souris et des métastases du foie chez neuf (20%) d'entre elles. Cette technique offre un modèle exact du CCR avec les tumeurs se développant dans le microenvironnement correct et se métastasant au niveau du foie avec une fréquence semblable à celle observée chez des patients présentant un cancer colorectal. La bioluminescente sérielle réduit quant à elle le nombre de souris nécessaires dans les études en éliminant la nécessité d'abattage pour évaluer du fardeau de la maladie.

## Abstract (de)

Die Verbesserung der Ergebnisse bei Darmkrebs (CRC) erfordert eine genauere in vivo-Modellierung der Erkrankung beim Menschen, um eine zuverlässigere präklinische Beurteilung potenzieller Therapien zu ermöglichen. Es sind neuartige bildgebende Verfahren notwendig, um die longitudinale Bewertung der Krankheitslast in diesen Modellen zu verbessern und die Anzahl der für translationale Studien benötigten Tiere zu reduzieren. Dieser Bericht beschreibt die Entwicklung eines immunkompetenten syngenetischen orthotopen CRC-Mausmodells unter Verwendung der Zäkumimplantation von CT26-Zellen, die stabil mit dem Luciferase-Gen in immunkompetente BALB/c-Mäuse transfiziert wurden, um eine serielle biolumineszente Darstellung des Krebsverlaufs zu ermöglichen. Die Lumineszenz in der stabil transfizierten CT26-Zelllinie spiegelte nach der Vorkonditionierung in der Flanke einer BALB/c-Maus die Zelllebensfähigkeit exakt wider und führte in der ersten Pilotstudie nach der Zäkuminjektion bei 5 von 8 (63%) Mäusen zu primären Zäkumtumoren. Das Leuchtsignal nahm während des gesamten Untersuchungszeitraums weiter zu, wobei eine Maus (20%) eine Lebermetastase entwickelte. Die histopathologische Beurteilung bestätigte, dass die Tumore mit einem schlecht differenzierten Adenokarzinom übereinstimmen. Wir haben diese Technik nun bei 68 immunkompetenten BALB/c-Mäusen durchgeführt. Es gab weder Komplikationen durch das Verfahren noch perioperative Todesfälle, wobei sich bei 44 (65%) Mäusen Primärtumore und bei neun (20%) von diesen Lebermetastasen entwickelten. Diese Technik bietet ein genaues CRC-Modell mit Tumoren, die sich in der richtigen Mikroumgebung entwickeln und in der Leber mit einer ähnlichen Häufigkeit metastasieren wie bei Patienten mit CRC, wobei die serielle Biolumineszenz die für Studien benötigte Zahl von Mäusen reduziert, da die Notwendigkeit der Tötung zur Beurteilung der Krankheitslast entfällt.

## Resumen

Mejorar los resultados para el cáncer colorrectal requiere una creación más precisa en vivo de modelos de la enfermedad en humanos, lo que permitiría una valoración preclínica más fiable para posibles terapias. Son necesarias nuevas técnicas de creación de imágenes para mejorar la evaluación longitudinal de la carga de la enfermedad en estos modelos, reduciendo el número de animales requeridos para estudios translacionales. Este informe describe el desarrollo de un modelo de murino singenéico ortotópico inmuno competente con un cáncer colorrectal, utilizando la implantación cecal de células CT26 transfectadas establemente con el gen luciferasa en ratones BABB/c inmuno competentes, permitiendo unas imágenes biuluminescentes en serie del avance del cáncer. La luminiscencia en la línea de células CT26 transfectadas, después de un preacondicionamiento en el blanco de un ratón BALB/c, reflejó de forma precisa la viabilidad de las células y provocó tumores cecales primarios en 5 de 8 (63%) de los ratones en el estudio piloto inicial después de una inyección cecal. La señal luminiscente continuó aumentando durante el periodo de estudio con un ratón (20%) desarrollando una metástasis hepática. La evaluación histopatológica confirmó que había tumores consistentes con un adenocarcinoma indebidamente diferenciado. Ahora hemos realizado esta técnica con 68 ratones BALB/c inmuno competentes. No ha habido ninguna compliación con el procedimiento o las muertes perioperativas, con un desarrollo principal de tumores en 44 (65%) de ratones y metástasis hepáticas en nueve (20%) de estos. Esta técnica aporta un modelo preciso de cáncer colorrectar con tumores desarrollándose en el microambinte adecuado y con metástasis hepática con una frecuencia similar a la que se ve en pacientes con cáncer colorrectal, con una bioluminiscencia serial que reduce los números de murinos requeridos en los estudios para eliminar la necesidad de sacrificar demasiados ejemplares para la realización de estudios.

## Introduction

The ideal murine model would provide an exact recapitulation of human colorectal cancer (CRC). It should develop spontaneously throughout the colon and rectum, have a high incidence in the animals with a short latency period, follow the same metastatic pattern, occur in immune-competent animals, allow non-invasive monitoring of disease progression and have the same molecular characteristics as the human disease. Current animal models fail to fulfil all these criteria, but with advanced imaging modalities, numerous cell lines and improved transgenic modelling methods available, there is a growing choice. The conversion of potential therapies to clinical practice requires selection and development of the correct murine model for the required application.

Before embarking on any study involving the use of animals, it is essential to understand the merits and limitations of available models; the authors spent considerable time researching current murine models of CRC with their findings published in a review article. This explains the importance of selecting the correct model for the required application to maximise the translational potential. The carcinogen-induced and genetically engineered models are useful in studies of the development and prevention of CRC, whereas tumour implantation models are preferred for screening candidate therapeutics, with subcutaneous implantation allowing high-throughput assessment for verification in orthotopic models.^1^

For the pre-clinical study of potential novel therapies in the treatment of CRC, ideally a tumour is required that grows in the correct microenvironment with the potential to metastasise, particularly to the liver, to increase the chance of translation to clinical practice. Imaging assessment of disease burden should allow data acquisition from several animals with minimal distress, in a short time frame and at low cost. It was felt the orthotopic implantation of a luminescent murine colorectal cancer cell line in an immune-competent animal best fulfilled these criteria. Developments in bioluminescent imaging (BLI) have allowed the detection of small numbers of cells in rodents and facilitated the assessment of disease burden without necropsy, resulting in a reduction in the number of mice required in experimental studies. BLI has some advantages over fluorescence imaging (FI) in animal studies as the background signal in FI can limit the detection of low cell numbers.^2^ BLI also allows detection of signal in a number of animals simultaneously, decreasing costs and the time required for data acquisition when compared with Computerised Tomography (CT) or Magnetic Resonance Imaging (MRI) assessment. The main limitation of BLI is light quenching, significant light absorption can occur as a result of the melanin found in the skin of pigmented mice. Animal fur can also scatter light and attenuate signal and therefore shaving is required in furred animals.^3^ It is also considered a semi-quantitative technique when compared to three-dimensional (3D) modalities but is well accepted in monitoring changes in tumour burden.

BLI relies on the induced expression of the foreign protein luciferase, which is not normally expressed in the cell of interest. Upon exposure to the luciferase enzyme, the substrate luciferin is oxidised to the excited-state molecule that emits light. This reaction requires oxygen and adenosine triphosphate and therefore is only possible in viable cells.^4^ The light emitted from the reaction can be detected using a luminometer or a cooled charge-couple device (CCD) camera.

This paper describes the development of a murine model of CRC through the caecal implantation of luminescent CRC cell lines, with the aim of allowing others to replicate this model for use in translational studies, maximising the chance of conversion to clinical trials in patients. The findings of an initial pilot study of orthotopic implantation are reported to guide researchers on expected tumour uptake and growth rates. It also highlights the use of BLI in reducing animal numbers, and the requirements for development and validation of luminescent cell lines, ensuring the acquisition of accurate data and describing methods to analyse and standardise findings.

## Materials and methods

### Luminescent vector

The pGL4.51[luc2/CMV/Neo] (Promega, Southampton, UK) vector was utilised for the creation of a luminescent cell line. The synthetic Neomycin resistance gene (G418) allows selection of successfully transfected cells and the ampicillin resistance gene is included for bacterial amplification of the vector.

### Transformation and amplification of vector

To increase the quantity of the vector, available plasmids were transformed and amplified in *Escherichia coli* bacteria. Four vials (50 µl per vial) of One Shot® TOP10 Chemically Competent *E. coli* bacteria (ThermoFisher Scientific, Paisley, UK) were thawed on ice. Under aseptic conditions, 1 µl of vector DNA was added to each vial, including the positive control DNA for transformation pUC19 (ThermoFisher Scientific, Paisley, UK, 10 pg/µl), which provides ampicillin resistance to transformed cells. Distilled water was added to the final vial as a negative control.

Vials were incubated on ice for 30 minutes and heated to 42℃ for 30 seconds. After a further 3 minutes on ice, 900 µl of pre-warmed Super Optimal broth with Catabolite repression (SOC) medium (ThermoFisher Scientific, Paisley, UK) was added. The contents of each vial were moved to 15 ml Falcon tubes (Fisher Scientific, Loughborough, UK) in a rocking incubator at 250 revolutions per minute (rpm) and 37℃ for one hour.

Luria Bertani (LB) broth-agar-ampicillin-coated 100 mm petri dishes (Greiner Bio-One, Stonehouse, UK) were made by combining 20 g LB powder (Sigma-Aldrich, Dorset, UK) with 15 g agar granules (Melford Laboratories Ltd., Ipswich, UK) in 1 L of sterile distilled water and adding ampicillin (Sigma-Aldrich, Dorset, UK) to a final concentration of 100 µg/ml. To ensure the development of individual well-spaced colonies, four different volumes of the contents from each falcon tube were spread over individual coated petri dishes and incubated at 37℃ overnight, a 10 µl filter tip was used to pick colonies. The 10 µl tip was placed directly into a LB broth-ampicillin mixture in a 15 ml Falcon tube and incubated overnight at 37℃. Once cloudy, the mixture was added to 1 L of LB broth (20 g LB powder in 1 L water) and incubated at 37℃ for 24 hours prior to the extraction of plasmid DNA.

### DNA/plasmid extraction from bacteria

Plasmid DNA was extracted and purified using the QIAGEN-tip 500 Plasmid Midi Kit (QIAGEN, Manchester, UK) as per the manufacturer's instructions. The concentration and purity of the plasmid DNA was determined by reference to the 260:280 nm ratio by placing 1 µl of the buffered DNA on the Nanodrop™ ND-1000 UV spectrophotometer (Labtech International, East Sussex, UK) as per the manufacturer's instructions. Only samples with a ratio ≥ 1.8 were used in subsequent experiments.

### Cell culture

Murine (CT26) CRC cell lines were purchased from the American Type Culture Collection. The CT26 cell line was developed from a colonic carcinoma induced by the administration of the carcinogen N-nitroso-N-methylurethane to BALB/c mice.^5^ CT26 cells were cultured in Roswell Park Memorial Institute (RPMI) 1640 medium (Sigma-Aldrich, Dorset, UK) at 37℃ with 5% CO_2_. Media were supplemented with 10% heat-inactivated foetal bovine serum, 584 mg/L L-glutamine, 100 units/ml penicillin G and 100 µg/ml streptomycin (Sigma-Aldrich, Dorset, UK). Transfected luminescent cells were co-cultured with G-418 (Promega, Southampton, UK).

When a specific number of cells were required, they were counted using 0.4% trypan blue solution and the Countess™ automated cell counter (Invitrogen, Paisley, UK). An equal volume of trypan blue and the cell-suspension were mixed and 10 µl injected into the slide.

### Determining lethal concentrations of selection antibiotics in cells

To allow antibiotic selection of transfected cells, the concentration of G418 required to kill all cells in a week was determined, cells were plated out at 1 × 10^5^ cells per well in 500 µl of medium on a 24-well culture plate (Nalge-Nuc international, C/O VWR International, Lutterworth, UK) for assessment of cell viability using trypan blue and the Countess™ automated cell counter.

Cells were dosed across a range of G418 concentrations from 0 to 1000 µg/ml in triplicate by dissolving the stock solution (1 mg/ml) in the appropriate volume of growth medium. Media, with the selection antibiotic, were changed every 48 hours.

### Transfection of cells with luminescent vectors

For the stable incorporation of luciferase expression into CT26 cells, Lipofectamine® 2000 (Invitrogen, Paisley, UK) was utilised as per the manufacturer's instructions. Cells were plated at 1 × 10^5^ cells per well in 500 µl of complete medium on a 24-well plate. Lipofectamine® 2000 was diluted in Opti-MEM Reduced Serum Media (Invitrogen, Paisley, UK) in sterile Eppendorf (Eppendorf UK Limited, Stevenage, UK) at four volume/volume (v/v) ratios. Next, 5 µg of plasmid DNA was also diluted in 250 µl of Opti-mem before 50 µl of the diluted DNA was mixed with 50 µl of the four diluted Lipofectamine® 2000 mixtures. Following 15-minute incubation at room temperature, the resulting four DNA-lipid complexes were added in duplicate to the medium on the cultured cells. As a Lipofectamine® 2000-only control, 5 µl of the transfection reagent was added to 100 µl of Opti-MEM and 50 µl added to duplicate wells of the 24-well plate. Cells were incubated at 37℃ for 48 hours before the assessment of luminescence.

### Assessment of luminescence by the Bright-Glo™ Assay

The Bright-Glo™ Luciferase Assay System (Promega, Southampton, UK) was one of the methods used to assess luminescence in cell lines. To assess the optimum transfection conditions, cells from one well of each pair of duplicates were lysed by aspirating the medium and adding 200 µl of the supplied lysis buffer. The plate was rocked at 70 rpm for 5 minutes and lysis buffer was aspirated from each well. Two 1:2 dilutions of the buffer were performed before 80 µl of each dilution was added to a separate well of a 96-well white-backed plate (Greiner Bio-One, Stonehouse, UK). Then 20 µl of the Bright-Glo™ reagent was added to each well, the plate spun at 700 rpm for 15 seconds and luminescence assessed on the Varioskan™ Flash Multimode Reader with an open filter, with luminescence normalised to the protein content in each well. The Pierce™ BCA Protein Assay Kit (ThermoFisher Scientific, Paisley, UK) was used for protein quantification.^6^

### Assessment of luminescence with in vivo grade luciferin

For the assessment of luminescence without cell lysis, *in vivo* grade luciferin was applied to cells in culture. Cells were seeded on 96-well plates using 10 1:2 dilutions starting at 1 × 10^4^ cells per well and left overnight to adhere. A stock solution of VivoGlo™ firefly luciferin (Promega, Southampton, UK) was created by diluting the powder to 15 mg/ml in 1x phosphate buffered saline (PBS) (Invitrogen, Paisley, UK). This stock solution was further diluted to 150 µg/ml in growth medium and 100 µl added to cells in culture on a 96-well white-backed plate for assessment in the Varioskan™ Flash Multimode Reader or on a clear plastic plate for the IVIS® Spectrum *in vivo* imaging system (Perkin-Elmer, Massachusetts, USA). For plate imaging in the IVIS® the stage was set to position C with the default settings of auto-exposure, medium binning and an open filter.

To establish the optimum imaging time after the application of luciferin to cells, plates were imaged until peak signal was obtained and subsequent imaging performed at this time point.

### Clonal selection and expansion of cell lines

Once a sufficient population of luminescent cells was available, clonal selection and expansion was performed through serial dilution in 96-well culture plates to a single cell per well. Then 200 µl of a cell suspension of 2 × 10^4^ cell per ml luminescent CT26 cells was added to the first well (A1). Next, 100 µl of culture medium was added to every other well on the plate before a 1:2 first dilution series was completed down the first column. An additional 100 µl of medium was added to each well in column one and a second 1:2 serial dilution performed, moving 100 µl from the first column to second, and this process repeated across the plate.

After 24 hours, wells were reviewed under x200 magnification and those with a single cell visible marked. After a further 72 hours marked wells were examined again and those with a single colony selected for serial passage and expansion under antibiotic selection.

### Murine studies

All animal experiments were performed in accordance with criteria outlined in the European Directive 2010/63/EU and in a Home Office UK approved project licence (PPL 70/8457) granted under the Animals (Scientific Procedures) Act 1986 and approved by the Animal Ethics Committee at the University of Liverpool. Male 6- to 8-week-old immune-competent BALB/cAnNCrl mice with a mean+/−SD weight of 23.8+/−0.3 g were purchased from Charles River Laboratories (Margate, UK) and housed in a licenced specified pathogen-free establishment. Male mice were used to allow easier access to the caecum on laparotomy. Mice were given free access to food (Special Diet Services CRM 9.5mm pelleted food, product code 801722) and water and housed at a temperature of 19–23℃ under a 12-hour light-dark cycle and a humidity of 50–60%. A maximum of five mice were housed in a single individually ventilated Techniplast GM500 (Buguggiate, Italy) cage with soft wood for nesting, enrichment material and translucent red nest domes. Animals were housed in groups to allow normal social interaction. Mice were allowed to acclimatise to their new environment for a minimum of 7 days prior to the commencement of experimental work. Twice-daily checks were routine in the unit, with mice involved in experiments checked a minimum of three times per day. As these were pilot studies designed to guide further experimental work, a limited number of mice were selected based on the predicting tumour uptake and growth rates to allow the calculation of sample sizes in future studies.

### Anaesthesia

For the induction of anaesthesia, mice were placed into an Evonik Plexiglas® anaesthetic chamber (Essen, Germany) and general anaesthesia (GA) induced with 3% isoflurane and an oxygen flow of 1 L per minute. Anaesthesia was confirmed by the absence of pedal withdrawal and animals were subsequently transferred to a nose cone and GA maintained on 1.5% isoflurane. Lubrithal™ gel (Northwich, UK) was applied to both eyes to prevent drying. Following experimental work mice were recovered on a heat pad or in a warming box until fully active, to prevent hypothermia.

### Subcutaneous injection of tumour cells

For subcutaneous (*sc*) flank injection, luminescent CT26 cells were suspended in a 1/1 (v/v) mixture of ice-cold PBS/Matrigel® (Corning, Amsterdam, The Netherlands), each mouse receiving 1 × 10^6^ cells in 100 µl. Prior to the injection of cells mice were anaesthetised as described previously in the manuscript, the right flank shaved and cells injected into the *sc* space using a 500 µl insulin syringe with a 30-gauge needle. Mice were ear-clipped for identification and tumour growth monitored using BLI as described below.

### Orthotopic caecal injection of tumour cells

For orthotopic injection, luminescent CT26 cells were suspended in PBS/Matrigel using the method described for *sc* injection. Mice were weighed and received a *sc* preoperative dose of buprenorphine (0.1 mg/kg). GA was induced and maintained by isoflurane using the same method described previously in the manuscript. Animals were transferred to a pre-warmed operating table, with GA maintained using a nose cone, and the abdomen shaved and prepped with betadine for disinfection. Aseptic technique and meticulous draping were used to maintain sterility of the operative field.

**Figure 1.**
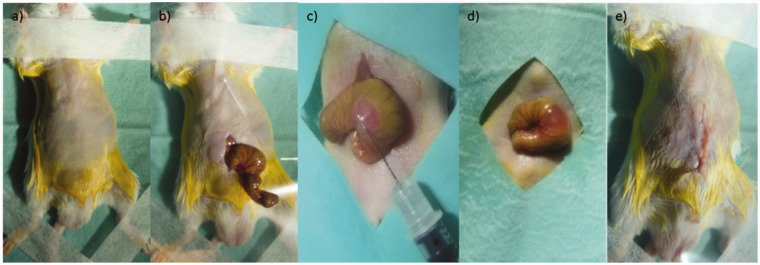
Technique for orthotopic injection of tumour cells. (a) Animals were placed in the supine position, the abdomen shaved, sterilised with betadine and sterile-draped. (b) A 1 cm lower midline laparotomy was performed, and the caecum delivered. (c) Tumour cells were injected into the subserosal plane, (d) ensuring a ‘bleb’ of cell suspension was achieved. (e) The wound was closed in two layers.

A 1–1.5 cm lower midline laparotomy was performed and moistened (sterile 0.9% saline) cotton tips used to deliver the caecum from the abdomen. A 500 µl insulin syringe with a 30-gauge needle was used to draw up 5 × 10^5^ cells in 50 µl of the PBS/Matrigel; it is important to note that after this pilot study the cell count was lowered to 4 × 10^5^ for subsequent experimental studies to prolong the study duration. After 45–60 seconds at room temperature, the entire volume was injected into the sub-serosal plane of the caecum under direct vision. The injection site on the caecum was standardised to the anti-mesenteric border of the anterior wall of the caecum. The needle entry site was gently compressed with a moistened cotton tip to prevent the leakage of cells and ensure haemostasis before returning the caecum to the abdomen. Peritoneum and muscle were closed with a continuous 6-0 Vicryl™ (Ethicon, subsidiary of Johnson & Johnson, Norderstedt, Germany) suture and skin using a subcuticular technique using the same suture (Figure 1).

Mice were ear-clipped for identification and allowed to recover in a warming chamber at 37℃. Additional buprenorphine analgesia was given, up to 0.05 mg/kg every 8 hours, if mice displayed pain-associated behaviours, such as a hunched posture. Only one mouse in the pilot study group required an additional dose of buprenorphine in the post-operative period, with normal behaviour exhibited shortly after surgery in the remaining seven. Tumour development, growth and the formation of metastases were monitored by bioluminescent imaging.

### Bioluminescent in vivo imaging

Mice were imaged in the IVIS® Spectrum *in vivo* imaging system after the intraperitoneal injection of luciferin. GA was induced using isoflurane, as described previously, and the area of interest shaved if fur had regrown since surgery. The IVIS® contains five nose cones on a warmed imaging platform for maintenance of GA during imaging.

Each mouse received a dose of 150 mg/kg VivoGlo™ firefly luciferin by injecting 10 μl/g bodyweight of the 15 mg/ml stock solution prior to transfer to the IVIS® imager. For mice flank-injected with tumour cells, imaging was conducted in the prone or left lateral position, whereas orthotopically injected mice were imaged supine. Imaging was conducted with an open filter, auto-exposure and medium binning. Mice were imaged on days 3, 7, 14 and 18 following the *sc* injection of tumour cells and on days 4, 7, 10, 14 and 17 following the caecel injection of tumour cells in these pilot studies. To establish an optimum imaging time following injection of luciferin, a kinetic-imaging curve was conducted. Mice were imaged every 2 minutes until the signal reached plateau.

To estimate signal depth and assess approximation to the liver, consistent with the development of metastasis, 3D tomographic reconstruction imaging by Diffuse Light Imaging Tomography was utilised. The animal was photographed by CCD camera and a 3D tomography constructed by the software. Luminescent signal was captured in five images over a range of spectral filters every 20 nm from 560 to 640 nm.

### Bioluminescent ex vivo imaging

To confirm liver lesions were consistent with metastases, organs were imaged *ex vivo*. After schedule 1 cull by cervical dislocation, although signal was in the plateau phase *in vivo* at 25 minutes post luciferin injection, necropsy was performed, caeca and livers with macroscopic disease were excised, placed in PBS and imaged with platform height set in position C, an open filter, auto-exposure and medium binning. The time between cull and *ex vivo* imaging was kept to the minimum, and did not exceed 3 minutes, to ensure signal was still present in excised tissue.

### Histological assessment and immunohistochemistry

After schedule 1 cull of mice by cervical dislocation, tissues were fixed in 4% paraformaldehyde (Sigma-Aldrich, Dorset, UK) and paraffin-embedded. Then 5 µm slices were cut on a rocking microtome, haematoxylin and eosin stained and examined by a veterinary pathologist (author LR) to confirm the presence of adenocarcinoma.

### Data analysis for luminescent imaging

Using the Living Image 4.1™ software, areas of luminescent signal were marked as regions of interest (ROI). In cell-based experiments, a 96-well plate-grid was placed over the image of the plate. To ensure reproducibility across time points in animal studies, the same size area was used to cover the abdominal area of each mouse. The number of photons per second within each marked ROI was calculated (total flux), background luminescent signal for each image was subtracted from the ROI value. A well containing cells and medium without luciferin allowed calculation of background signal, whereas in animal studies background luminescence was calculated by placing an ROI on an area of a mouse without signal. To allow each mouse to act as its own control luminescence was expressed as a fold-change from the initial reading. Graphical display and statistical analysis of data was performed using Prism® 7 (GraphPad Software, California, USA).

## Results

### Cytotoxicity of G418

Incubation of CT26 cells with G418 resulted in cell death in a dose-dependent manner with cell death achieved at concentrations of G418 ≥ 600 µg/ml. CT26 cells transfected with the vector pGL4.51 were cultured with 600 µg/ml of G418 in medium.

### Assessment of optimum transfection conditions

Luminescence was assessed using the Bright-Glo™ Assay in the CT26 cell line 48 hours after transfection with pGL4.51 plasmid DNA. The replicate well of the transfection conditions resulting in the highest luminescent signal per µg of protein were labelled CT26luc and passaged.

### Clonal selection and expansion

After serial dilution of the CT26luc population, which produced seven photons per second per cell, analysis of luminescence in the 13 clonal populations was performed on both the IVIS® and Varioskan™ after the application of VivoGlo™ firefly Luciferin. One clone (CT26lucA6) demonstrated strong luminescent signal and was expanded in culture, producing 27 photons per second per cell.

### Optimum imaging time with VivoGlo™ firefly luciferin

**Figure 2.**
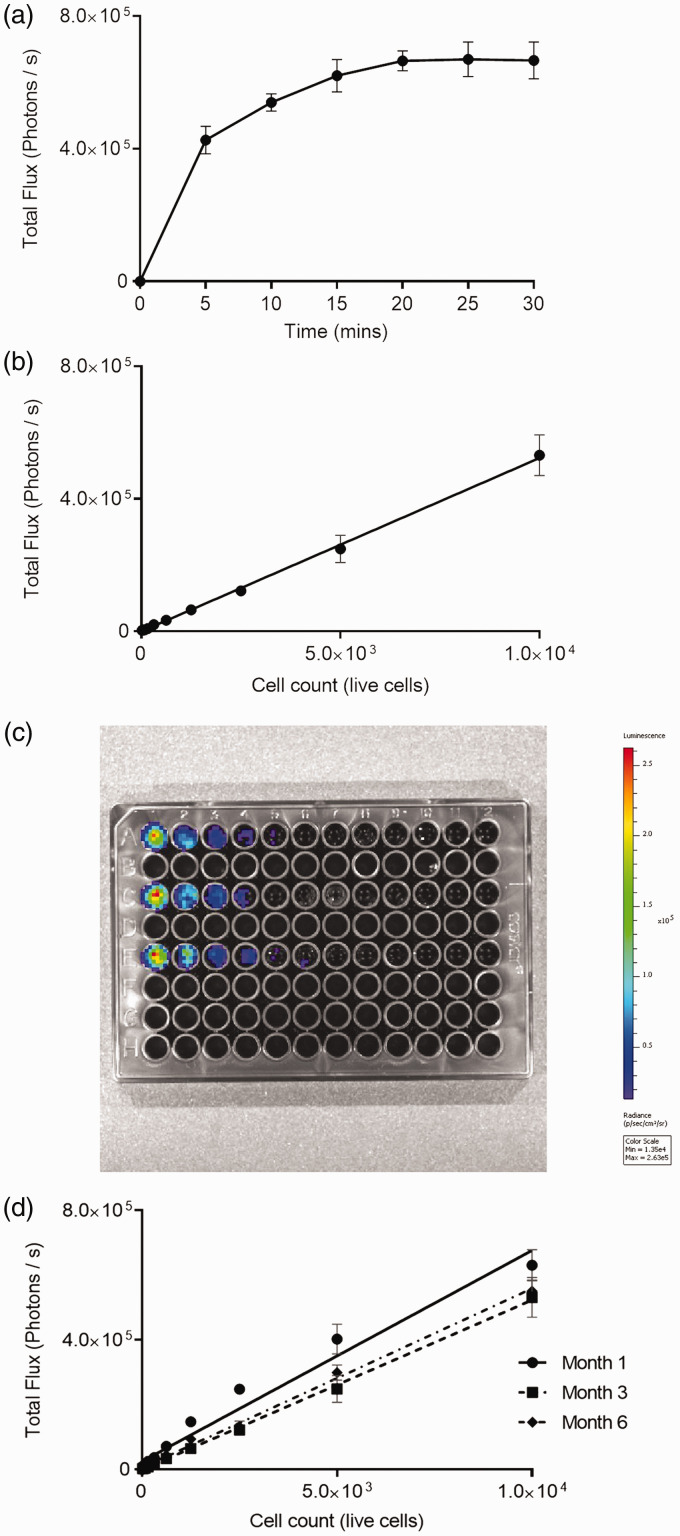
*In vitro* assessment of CT26lucA6 cells. (a) Kinetic imaging assessment of luminescence in CT26lucA6 cells using IVIS® imaging after the application of in VivoGlo™ firefly luciferin. This curve was generated to guide the timing of imaging after the application of luciferin in subsequent experiments. (*n* = 3 in triplicate with graph displaying mean +/− SD, min = minutes). (b) Graph demonstrating positive correlation between luminescence and the live cell count per well in CT26lucA6 cells (r^2^ = 0.98, *p* < 0.0001, Pearson R). (c) Image of a 96-well plate demonstrating fall in luminescent signal with decreasing numbers of CT26lucA6 cells. (d) Graphs display stability of luminescent signal in the CT26lucA6c after increasing time in culture.

To establish an optimum imaging time after the application of VivoGlo™ firefly luciferin to cells, a kinetic imaging curve was performed. Peak signal was achieved by 20 minutes; subsequent imaging with VivoGlo™ firefly luciferin was therefore conducted at this point (Figure 2(a)).

### Luminescence signal reflects the live cell count

To ensure luminescent signal was an accurate reflection of the number of viable cells present, luminescence was plotted against cell count. There was a strong positive correlation between cell count and luminescent signal and clones tested (r^2^ = 0.98, *p* < 0.0001) (Figure 2(b) and (c)).

### Luminescent stability in cell lines

To assess the stability of luminescence in clonal populations across passages, repeated IVIS® imaging was undertaken 1 month, 3 months and 6 months after the development of clones. Differences in luminescent signal were minimal across these time points, despite increasing passage number and freeze-thawing (Figure 2(d)).

### Subcutaneous injection of the CT26lucA6 clone

**Figure 3.**
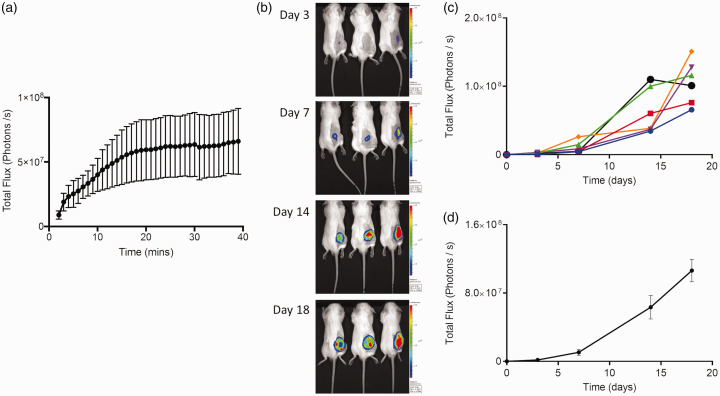
Subcutaneous grafting of the CT26lucA6 cell line in BALB/c mice. (a) Kinetic imaging curves from three BALB/c mice 14 days after the flank injection of CT26lucA6 cells, displaying total flux from the tumour+/− standard error in the mean (SEM) against time after the injection of luciferin. (b) Representative images of three BALB/c immune-competent mice serially imaged in the IVIS® after the subcutaneous (*sc*) injection of CT26A6 cells. (c) Luminescence signal increased throughout the study period in an exponential manner, as displayed for individual mice in a graph of time versus luminescence. (d) When data were combined the SEM was wide, reflecting the variable growth rates and therefore luminescent signal in mice (*n* = 6, graph displays mean +/− SEM).

To ensure cells would propagate *in vivo*, and assess the intensity and stability of luminescence, CT26lucA6 cells were injected into the right flank of 6 BALB/c immune-competent mice. A kinetic imaging curve was performed in the IVIS® at each imaging time point until day 14, these consistently demonstrated a plateau in signal intensity from 20–30 minutes, with mice imaged within this time window in subsequent studies utilising the flank grafting of cells (Figure 3).

The tumour uptake rate was 100% and luminescent signal increased throughout the imaging period as the tumour increased in size until imaging at day 18, where one tumour demonstrated some decrease in luminescence, presumably due to tumour necrosis. Mice were culled on the 18^th^ day as tumours reached the size stipulated in the project licence; this rapid development of tumours meant a lower cell count of 5 × 10^5^ cells in 50 µl was injected in future studies.

At the end of the study, tumours were excised after schedule 1 cull and the largest dissected with a scalpel blade and individual tumour lumps placed in wells of a six-well culture plate. RPMI 1640 medium, with the selection antibiotic G418, was added and after 3 days the tumour lumps were removed, leaving the adherent tumour cells in culture. This allowed the development of an *in vivo*-conditioned cell line for caecal implantation, labelled CT26lucA6c.

### Caecal implantation of the CT2luc6A6c cell line

**Figure 4.**
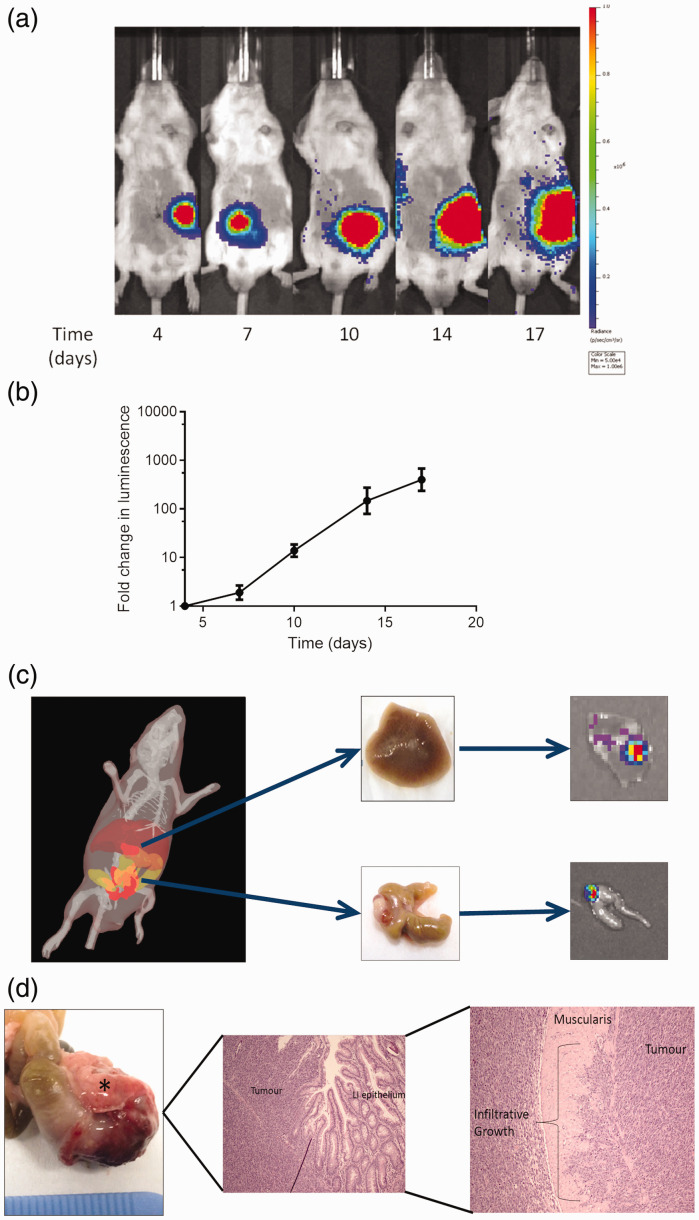
Development of the syngeneic orthotopic model. (a) Representative IVIS® images following the caecal injection of CT26lucA6c cells in to BALB/c mice. Imaging was consistent with development of a primary tumour in the left iliac fossa of the mouse, with ectopic signal developing on day 17 in the right upper quadrant, consistent with a liver metastasis. (b) Results were combined as fold-change in luminescence for graphical display, confirming an increase in luminescence throughout the study period. (*n* = 5, graph displays mean +/− SEM). (c) Three-dimensional spectral un-mixing imaging can be useful for estimating the depth of luminescent signal within the mouse, suggesting luminescent signal arising from within the liver. Ex-vivo assessment confirmed the presence of a liver metastasis, with signal present in both the caecum and liver on IVIS® imaging. (d) Photograph and histology from a caecal carcinoma (marked by an asterisk (*)) excised 18 days after implantation. Histology confirmed tumour growth in the wall of the caecum, originating below the epithelium and invading into the muscularis. LI: lower intestine.

The CT26lucA6c conditioned cell line was used to develop the syngeneic orthotopic murine model. To establish optimum imaging times, growth rate of primary tumours and frequency of metastatic disease, eight immune-competent BALB/c mice underwent orthotopic injection of the CT26lucAc6 cell line in the caecal sub-serosa. Primary tumours developed in five (63%) mice, with luminescent signal continuing to increase throughout the study period (Figure 4(a)).

Due to the variation in signal at the first imaging point, subsequent luminescent values were expressed as the fold-change in luminescence. The study was ended on the 18th day post-implantation due to one mouse nearing the severity limits of the project licence due to symptoms of obstruction (Figure 4(b)).

One (20%) mouse developed ectopic signal consistent with the development of a liver metastasis and was subject to 3D imaging, which suggested that signal was present within the liver parenchyma. This was confirmed on necropsy, ex-vivo imaging and histologically. Mice with luminescent signal all had macroscopic primary tumours at necropsy. Primary tumours were confirmed to be poorly differentiated neoplastic growths of epithelial origin, displaying clear infiltrative behaviour (Figure 4(c) and (d)).

Establishing an optimum time for imaging after the injection of luciferin was more difficult in the orthotopic model. The timing of peak signal tended to vary between mice (ranging from 4–25 minutes) and the plateau period short, reflecting variations in blood supply to the tumour. Mice were therefore imaged every 2 minutes until luminescent signal fell and the highest signal used for analysis at each time point throughout the study.

Our group have performed the orthotopic implantation of CT26lucA6c cells on a total 68 immune-competent BALB/c mice. There has been no peri-operative morbidity or mortality. Primary tumours have developed in 44 (65%) mice and of these nine (20%) have developed liver metastases.

## Discussion and conclusions

This paper describes the development of a syngeneic orthotopic metastatic murine model of CRC. This pilot study of the model allowed improvement of the injection technique, the establishment of imaging protocols and the determination of the ideal imaging frequency in addition to the tumour uptake rate, frequency of metastatic disease and duration of murine studies. As a direct, result future studies utilising the model followed a set protocol that included: the use of a lower cell count (4 × 10^5^ cells), allowing the study period to be prolonged to 21 days; the warming of Matrigel-suspended cells at room temperature for 1 minute to increase the viscosity; the use of a fine insulin needle to prevent peritonitis in the event of accidental full-thickness puncture of the caecum; the use of the 7th day post-implantation as the first imaging time point, giving time for the tumour to become established, but reducing the variations in signal seen between mice at later imaging points; the use of kinetic imaging, improving comparisons between time points; and the expression of data as fold-changes from the initial luminescent imaging point, allowing each mouse to act as its own control. Imaging frequency was also increased to three times a week for murine experiments comparing treatment groups due to the rapid growth of tumours.

A distinct advantage of this syngeneic model is the short time-frame required for the establishment and development of primary tumours, allowing rapid screening of potential therapies. This contrasts the results demonstrated in publications utilising orthotopic xenografting of human CRC cell lines. In one example 2 × 10^6^ cells were injected into the caecal wall and tumour growth assessed 8 weeks after implantation.^7^ The use of BLI imaging also increased throughput as five mice could be imaged simultaneously.

The rate of liver metastases (20%) demonstrated in the model is similar to the incidence of synchronous liver metastasis seen in patients with CRC,^8^ suggesting it is a reasonable representation of the presentation of CRC in patients, but limiting its use in studies where liver metastases are a requirement. In this scenario, direct injection into the liver parenchyma, portal vein or the spleen is likely to be more reproducible.^9^ A metastatic rate of 20% is lower than demonstrated in some studies; a recent publication demonstrating liver metastases in 67% of mice 14 days after caecal injection of CT26 cells. This study utilised MRI imaging, which could have improved detection rates, on a small number of mice (*n* = 6).^10^ MRI imaging was considered, but BLI imaging selected due to its high throughput and lower cost.

Another limitation of the model is the late detection of metastases, often only visualised separate from the primary tumour towards the end of the study period. Development of cells with greater luminescence could potentially facilitate the earlier detection of metastases. Other techniques have been described in the development of luminescent cell lines. It is possible to design vectors that allow cells to be dual-tagged with genes coding for luciferase and green fluorescence protein under the control of a single promotor, permitting selection of the successfully transfected luminescent cells by fluorescence-activated cell sorting, removing the need for serial dilution to randomly select clones.^11,12^ It is also possible that the dominant signal from the primary tumour masks that from liver metastases, if early detection of metastases is required for experimental data then covering the primary with a material of low auto-luminescence, such as black card, followed by the use of long exposure times could overcome this. Other imaging techniques, such as MRI, could then be considered if this fails to allow early detection of metastases.

Alternate transfection techniques could also have been considered. Comparisons of commercially available non-viral transfer reagents demonstrated cell line-dependent variations in transfection efficiency,^13^ whereas transfection techniques have also been compared in the literature; in human dental follicle cells the use of electroporation resulted in a greater transfection efficiency than chemical techniques.^14^ Viral transfection methods could also be considered; lentiviruses are well studied and demonstrate good integration in to the genome. As with all gene transfer methods there are drawbacks; production is often labour intensive and there is the potential for the activation of latent disease, particularly with retroviral vectors.^15,16^ Clustered, regularly interspaced short palindromic repeats gene editing is a relatively new technique, allowing a cell's genome to be cut at a desired location and a new gene to be inserted.^17^ Using this to splice the luciferase gene into the genome of a cell could have reduced the problems associated with poor transfection efficiencies and stability. Luminescent CT26 cell lines are now commercially available with varying luminescent signal. However, in-house development of a population from a single stable luminescent clone increased confidence in our data and improved reliability across experimental repeats.

The aim of this work was to create a murine model of CRC that more accurately reflected the pattern of disease found in patients than with the *sc* flank injection of tumour cells into rodents. This model has some distinct advantages for the testing of potential therapies: the use of BLI imaging as a refinement to the model allows longitudinal data acquisition, with cull not required for the assessment of disease burden, significantly reducing the number of animals required for experiments; the rapid development of disease means studies can be conducted over short time periods, minimising any impact on animal welfare; and the use of immune-competent mice minimises costs in comparison to immune-deficient models, in addition to allowing the study of the tumour immune microenvironment and facilitating the testing of immune therapies.

Although this model has been reported previously,^18^ this study provides a detailed description of the methods required to develop the model and allow BLI assessment of disease burden, from the transfection and validation of cell lines to the techniques required for caecal implantation. It provides researchers with an accurate representation of expected tumour uptake and growth rates, guiding future experimental work. Importantly, this study highlights the need for small-scale pilot studies prior to the acquisition of experimental data, the data from which would allow refinements of technique and accurate sample size calculations, increasing the likelihood of achieving statistically significant data while ensuring a minimum number of animals are utilised.

If we are to increase the translational of potential therapeutics from bench to bedside, then accurate animal modelling is required. The orthotopic murine model of CRC described in this study offers one of the most realistic representations of the presentation of CRC in humans, while allowing studies to be conducted in enough animals and in a short enough time frame to be both cost and time effective.
